# Bronchoalveolar lavage fluid and serum 1,3-β-d-glucan testing for invasive pulmonary aspergillosis diagnosis in hematological patients: the role of factors affecting assay performance

**DOI:** 10.1038/s41598-020-75132-3

**Published:** 2020-10-21

**Authors:** Barbora Weinbergerova, Tomas Kabut, Iva Kocmanova, Martina Lengerova, Zdenek Pospisil, Zdenek Kral, Jiri Mayer

**Affiliations:** 1 Department of Internal Medicine-Hematology and Oncology, Masaryk University and University Hospital, Brno, Czech Republic; 2 Department of Clinical Microbiology, University Hospital, Brno, Czech Republic; 3 CEITEC – Central European Institute of Technology, Masaryk University, Brno, Czech Republic; 4 Department of Mathematics and Statistics, Faculty of Science, Masaryk University, Brno, Czech Republic

**Keywords:** Biomarkers, Diseases, Oncology

## Abstract

Invasive fungal disease (IFD) early diagnosis improves hematological patient survival. Non-culture-based methods may reduce diagnostic time to identify IFD. As complex data on the value of 1,3-β-d-glucan (BDG) from bronchoalveolar lavage fluid (BALF) compared to serum for the most frequent invasive pulmonary aspergillosis (IPA) diagnosis are scarce, particularly including evaluation of potential factors adversely affecting BDG assay, we provided prospective single-center analysis evaluating 172 episodes of pulmonary infiltrates with BDG detection in BALF and serum samples collected in parallel among hematological patients from 2006 to 2015. Proven and probable IPA were documented in 13.4% of the episodes. Sensitivity (SEN), specificity (SPE), positive and negative predictive value (PPV; NPV), and diagnostic odds ratio (DOR) of the BDG assay using standard (80 pg/ml) cut-off for BALF were: 56.5%; 83.2%; 34.2%; 92.5%, and 6.5, respectively, and for serum were: 56.5%; 82.6%; 33.3%; 92.5%, and 6.2, respectively. The same BDG assay parameters employing a calculated optimal cut-off for BALF (39 pg/ml) were: 78.3%; 72.5%; 30.5%; 95.6%, and 9.5, respectively; and for serum (40 pg/ml) were: 73.9%; 69.1%; 27.0%; 94.5%, and 6.3, respectively. While identifying acceptable SEN, SPE, and DOR, yet low PPV of both BALF and serum BDG assay for IPA diagnosis, neither the combination of both materials nor the new optimal BDG cut-off led to significant test quality improvement. Absolute neutrophil count and aspirated BALF volume with a significant trend affected BDG assay performance. The BDG test did not outperform galactomannan assay.

## Introduction

Invasive fungal diseases (IFD) are life-threatening infections in patients with hematological malignancies^1–3^. Pulmonary infiltrates could represent an IFD warning sign, and differential diagnosis is crucial for the early start of preemptive antibiotic and antifungal therapy^4–7^.

Non-culture-based methods with their high sensitivity (SEN) and specificity (SPE) reduce diagnostic time to identify IFD. 1,3-β-d-glucan (BDG), a major cell wall component of most fungal species, is released into blood and tissues during IFD (except mucormycetes and *Cryptococcus* spp.). The Fungitell test is the only BDG antigenemia assay recommended^4,5,8–10^. Galactomannan (GM) is well established as a reliable BALF and serum marker in early detection of invasive aspergillosis^4,5,9,11–13^.

Several studies and meta-analyses concerning serum BDG assay performance in IFD diagnosis with variable outcomes have been provided^8,14–21^. Data regarding BDG from bronchoalveolar lavage fluid (BALF) for differential diagnosis of pulmonary infiltrates exists although limited and heterogeneous^22–26^. Heterogeneity is caused by various factors and lacks a complex analysis. Data is also insufficient regarding added value of a BDG combination from serum and BALF^23–26^.

We therefore recognized the need to evaluate and reconsider the role of BDG from serum and BALF in detection of early invasive pulmonary aspergillosis (IPA) the most common IFD. Reassessing BDG detection accuracy regarding a commonly employed 80 pg/ml cut-off, we implemented superior measures to propose a new optimal BDG cut-off value in serum and BALF samples for IPA diagnosis. Our secondary goal was to analyze defined clinical factor impact on test accuracy with a standard of 80 pg/ml cut-off. Furthermore, we sought to identify and qualify increased IPA detection accuracy when serum and BALF samples were obtained in parallel.

## Methods

### Study population

Our prospective cohort study involved consecutive non-selected hematological patients treated at our department from 2006 to 2015. We reviewed database clinical and laboratory records concerning epidemiology, diagnosis, and therapy of patients who underwent bronchoscopy with BALF and serum testing for both BDG and GM at exactly the same time to evaluate pulmonary infiltrates on chest high resolution computed tomography (HRCT). Patients with repeated sampling were included in the analysis if the BAL was performed on a clearly new presentation of pulmonary infiltrate evaluated by an expert radiologist. Episodes with apparent false-positive results for GM in the serum caused by the administration of GM-positive tested lots of piperacillin-tazobactam or Plasma-Lyte solution (Baxter Healthcare) were excluded from the analysis. The sample collection and research were approved by the Local Ethics Committee of the University Hospital Brno, Czech Republic, Number 01-170920/EK. All research was performed in accordance with relevant guidelines and regulations. Informed consent was obtained from all participants.

### Fiberoptic bronchoscopy and sample investigation

The fiberoptic bronchoscopy site was guided by the HRCT pathological finding. Eight to 10 sequential, 20-ml aliquots of sterile saline solution were infused into the lower respiratory tract, and each aliquot was immediately aspirated. The first bronchial sample aliquot's return was processed separately from subsequent aliquot returns, which were pooled together and homogenized (BALF). BALF was subjected to cytology assessment, direct examination, bacterial, fungal, mycobacterial culture; galactomannan and BDG detection; polymerase chain reaction (PCR) testing for *Aspergillus fumigatus*, mucormycetes, *Pneumocystis jirovecii*, and viral pathogens. Serum samples obtained simultaneously with bronchoscopic material were examined for GM and BDG detection, bacterial and fungal culture.

### 1,3-β-d-glucan detection

For BDG detection, a commercial kit (Fungitell, Associates of Cape Cod, Inc., Cape Cod, MA, USA) was used according to manufacturer’s instructions for sera samples and equally for BALF samples. BALF specimens were centrifuged at 1000 rpm for 10 min, and supernatant was used for BDG detection. Thereafter, the BALF samples were equally treated as sera samples. Samples were frozen at − 20 °C until BDG level determination. A positive test result was defined as a sample with cut-off level ≥ 80 pg/ml for both, serum and BALF. A detailed methodology of BDG assessment in serum and BALF has been previously described^27^.

### Case definition and important clinical parameters

Each case was classified as proven, probable, possible, or no IFD according to EORTC/MSG (European Organization for Research and Treatment of Cancer/Mycoses Study Group) criteria^4^, however Fungitell test results were not included as one of the microbiological criteria. Cases classified as proven and probable IFD were considered true-positives; no IFD as true-negatives. Eighty-three episodes with possible IFD, 8 episodes with invasive mucormycosis, one episode caused by *Alternaria* sp., and 18 *Pneumocystis* pneumonia episodes were not included in our final analysis.

We not only assessed BDG detection accuracy in BALF and serum with 80 pg/ml cut-off, moreover we sought to set up new accurate IFD detection cut-off levels based on sensitivity (SEN) and specificity (SPE) optimal combination. Parameters possibly affecting BDG assay were analyzed from BALF: Antifungals administration and its duration prior to sampling, concomitant *Candida* spp. positive culture from BALF or oral cavity, positive bacterial culture from BALF, absolute neutrophil value, and aspirated BALF volume; and from serum: Antifungals administration and its duration prior to sampling, concomitant bacteremia, and absolute neutrophil value. Furthermore, we compared BDG to GM test accuracy in both, BALF and serum, for IFD diagnosis.

### Statistical analysis

Continuous variables were compared using the Mann–Whitney U test and the Spearman correlation coefficient (R_S_). Categorical parameters relation was evaluated using Pearson’s Chi-squared and Kendall’s tau tests. For all analyses, α = 0.05 was used as a level of statistical significance. Sensitivity (SEN), specificity (SPE), negative predictive value (NPV), positive predictive value (PPV), diagnostic odds ratio (DOR), accuracy, were calculated for both serum and BALF BDG assay, and combination of both materials. A new BDG-max variable was evaluated based on the higher BDG value of the pair samples, BALF, and serum. A receiver operating characteristic (ROC) curve and area under the curve (AUC) were used to estimate BDG assay discriminatory capability performed in samples of BALF and serum for IFD detection. The influence of all monitored variables on IFD prediction was evaluated using a multidimensional logistic regression model. For statistical analysis, software R version 3.5.2 was used.

## Results

In total, 172 unique episodes (from 152 adult patients) were analyzed with characteristics described in Table 1. Proven, probable, and no IFDs were documented in 8 (5%), 15 (9%), and 149 (86%) episodes, respectively. Invasive aspergillosis was present in all eight proven and fifteen probable cases.

**Table 1:** Baseline characteristics of episodes with BDG detected in BALF and serum.

Total number of episodes	172
Total number of patients	152
Number of episodes per patient, median (range)	1 (1–3)
Age (years), median (range)	56 (18–78)
Sex, male, *n *(%)	96 (55.8)
**Definitive probability of pulmonary IFD according to EORTC/MSG criteria from 2008, n (%)**	
Proven IFD	8 (4.7)
Probable IFD	15 (8.7)
No IFD	149 (86.6)
**Underlying disease and anticancer therapy at baseline, n (%)**	
AML + MDS	76 (44.2)
Induction/reinduction of AL	47 (27.3)
Allogeneic SCT	34 (19.8)
Antifungals at the time of sampling, *n *(%)	145 (84.3)
Fluconazole	54 (37.2)
Other (echinocandins, itraconazole, posaconazole, voriconazole, conventional and lipid-based amphotericin, and their combination)	91 (62.8)
Duration of antifungal therapy prior to sampling, median of days (range)	4 (0–540)
*Candida* spp. isolated from oral cavity, *n *(%)	55 (32.0)
*Candida* spp. isolated from BALF, *n *(%)	11 (6.4)
*Candida* spp. bloodstream infection, *n *(%)	0 (0)
Bacteria isolated from BALF, *n *(%)	25 (14.5)
Bacteremia at the time of sampling, *n *(%)	6 (3.5)
Aspirated BALF volume, median of ml (range)	80 (32–160)
Absolute neutrophil count × 10^9^/l at baseline, median (range)	0.4 (0.0–12.2)

BDG, 1,3-β-d-glucan; BALF, bronchoalveolar lavage fluid; IFD, invasive fungal disease; EORTC/MSG, European Organization for Research and Treatment of Cancer/Mycoses Study Group; AML, acute myeloid leukemia; MDS, myelodysplastic syndrom; AL, acute leukemia; SCT, stem cell transplantation; BALF; bronchoalveolar lavage fluid.

### BDG assay evaluated by a standard cut-off of 80 pg/ml

**Figure 1:**
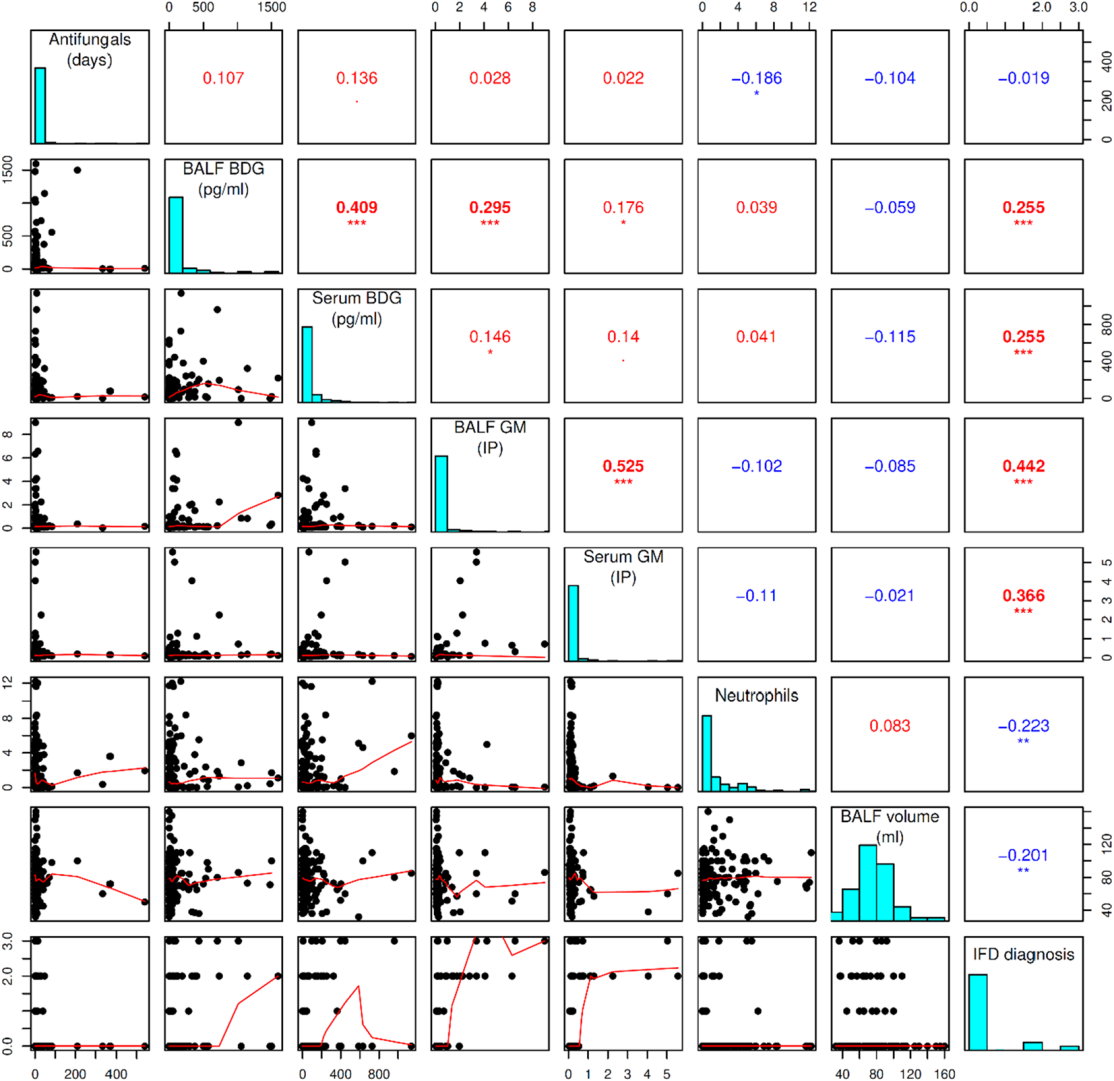
Correlation between continuous parameters possibly influencing the BDG assay quality in IFD diagnosing. In this figure shows Spearman correlation matrix representing the relation of continuous parameters evaluated by the Spearman coefficient. The diagonal from the upper left corner to the lower right corner contains frequency histograms of each variable. In the histogram “IFD diagnosis”, the episode numbers are divided according to the probability of IFD: 0—no IFD; 2—probable IFD; 3—proven IFD. On the bottom of the diagonal, there are the bivariate scatter dot plots with fitted lines displayed of the variable pairs. On the top of the diagonal, the pairwise correlations of variables (Spearman rank correlation coefficients; S) are with the significance level represented by stars: *p < 0.05; **p < 0.01; ***p < 0.001. BDG, 1,3-β-d-glucan; BALF, bronchoalveolar lavage fluid; IFD, invasive fungal disease; GM, galactomannan; BAL, bronchoalveolar lavage.

BDG values median in BALF and serum was 16.0 pg/ml (min-0; max-1594) and 22.0 pg/ml (min-0; max-1138), respectively. GM values median in BALF and serum was 0.13 IP (index of positivity) (min-0.04; max-1.98) and 0.13 IP (min-0.03; max-0.52), respectively. The correlation between BDG and GM levels in BALF and serum is indicated in Fig. 1.

Median BDG levels in proven and probable episodes versus no IFD were in BALF 111 versus 13 pg/ml and in serum 113 versus 20 pg/ml. The correlation between BALF or serum BDG levels and IFD probabilities (R_S_ = 0.255; and R_S_ = 0.255, respectively) appears in Fig. 1.

Based on ROC analysis, BDG assay performance for BALF and serum indicated SEN (56.5%; and 56.5%), PPV (34.2%; and 33.3%), AUC (0.671; and 0.677), SPE (83.2%; and 82.6%), NPV (92.5%; and 92.5%), and IFD prediction accuracy 0.8 with a standard cut-off of 80 pg/ml (Table 2). BDG-max assay affirmed SEN (65.2%), SPE (73.2%), and 0.6 diagnostic accuracy (Table 2).

**Table 2:** BDG performance assay for prediction of proven and probable IFD diagnosed according to EORTC/MSG criteria.

Cut-off (pg/ml)	SEN	SPE	PPV	NPV	DOR	Accuracy
**BALF BDG**						
80	0.565	0.832	0.342	0.925	6.5	0.797
39	0.783	0.725	0.305	0.956	9.5	0.733
**Serum BDG**						
80	0.565	0.826	0.333	0.925	6.2	0.791
40	0.739	0.691	0.270	0.945	6.3	0.698
**BDG-max**						
80	0.652	0.732	0.273	0.932	5.1	0.721
39/40^a^	0.870	0.567	0.235	0.966	7.9	0.607

BDG, 1,3-β-d-glucan; BALF, bronchoalveolar lavage fluid; IFD, invasive fungal disease; EORTC/MSG, European Organization for Research and Treatment of Cancer/Mycoses Study Group; SEN, sensitivity; SPE, specificity; PPV, positive predictive value; NPV, negative predictive value; DOR, diagnostic odds ratio.

^a^39 pg/ml cut-off for BALF and 40 pg/ml cut-off for serum.

### BDG assay evaluated by a new suggested optimal cut-off

Based on ROC analysis, our challenging task was to determine optimal BDG cut-off value in both serum and BALF. We identified new BDG cut-offs for both BALF (39 pg/ml) and serum (40 pg/ml). The new suggested BALF and serum value reached SEN (78.3% and 73.9%), SPE (72.5% and 69.1%), and DOR (9.5 and 6.3) (Table 2).

### Clinical factors possibly influencing BDG performance accuracy

**Figure 2:**
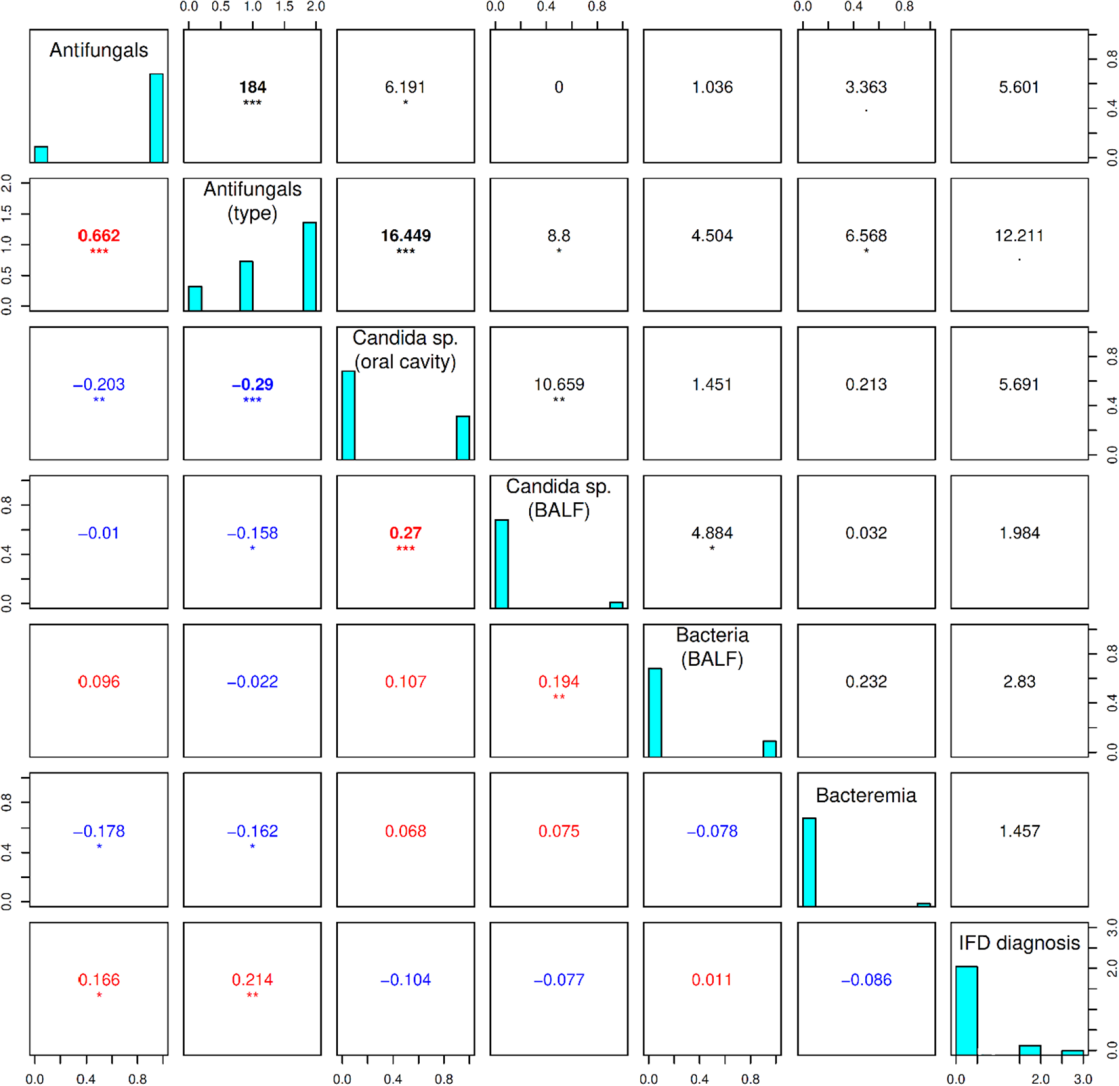
Correlation between categorical parameters evaluated by using the Pearson’s Chi-squared tests (upper right) and Kendall’s tau tests (lower left). In this figure represents the relation between categorical parameters. The diagonal from the upper left corner to the lower right corner contains frequency histograms of each variable. In the histogram “IFD diagnosis”, episode numbers are divided according to the probability of IFD: 0—no IFD; 2—probable IFD; 3—proven IFD. Concerning “Antifungals”: 0—no antifungals prior to sampling; 1—any antifungals prior to sampling; “Antifungals (type)”: 0—no antifungals; 1—fluconazole; 2—antifungals with broader spectrum; other histograms: 0—absent; 1—present. The Pearson’s Chi-squared tests (on the right top of the diagonal) measure the strength of a linear association between categorical variables presented by Pearson correlation coefficient (ρ) valued between + 1 and − 1, where 1 is a total positive linear correlation, 0 is no linear correlation, and − 1 is total negative linear correlation. The Kendall’s tau tests (on the bottom left of the diagonal) represent the ordinal dependence between two measured quantities based on the τ coefficient. Each significance level is represented by stars: *p < 0.05; **p < 0.01; ***p < 0.001. BDG, 1,3-β-d-glucan; BALF, bronchoalveolar lavage fluid; IFD, invasive fungal disease; GM, galactomannan; BAL, bronchoalveolar lavage.

We did not verify any significant relationship between probability of final IFD diagnosis and concomitant *Candida* spp. positive culture of oral cavity or BALF at time of sampling (ρ = 5.7, and 2.0; *p* > 0.05; respectively) (Fig. 2).

Similarly, the positivity of BALF bacterial culture and bacteremia did not suggest any significant correlation with IFD probability (ρ = 2.8, and 1.5, respectively; *p* > 0.05). (Fig. 2).

Significant correlation between lower absolute neutrophil value at the time of sampling and the higher degree of IFD probability was substantiated in our analysis (R_S_ = −0.223; *p* < 0.01) (Fig. 1).

More effective agents with a broader antifungal spectrum were more frequently administered at the time of BALF among episodes finally defined as proven or probable IFD with statistical significance (*p* < 0.01) (Fig. 2). Prolonged antifungals administration prior to sampling correlated with higher serum BDG levels (R_S_ = 0.136) (Fig. 1).

Eventually, episodes with higher aspirated BALF volume > 80 ml had a lower median of BALF-BDG value compared to episodes with lower aspirated BALF volume ≤ 80 ml (10 pg/ml vs. 21 pg/ml). Moreover, lower BALF BDG levels corresponded to higher aspirated BALF volume in the Spearman correlation test, yet results were not statistically significant (R_S_ = −0.059; *p* > 0.05) (Fig. 1).

### Logistic regression model evaluating clinical factors potentially influencing BDG and GM test accuracy

The model evaluating only BDG revealed an absolute number of neutrophils as a factor influencing BDG predictive value for IPA (Table 3). Higher absolute neutrophil count decreased BDG assay performance predictive value with statistical trend (*p* = 0.099). Additionally, we affirmed a trend in correlation between higher aspirated BALF volume and reduced opportunity to correctly predict IPA using BALF BDG (*p* = 0.085). No other factors significantly affected BDG assay prediction for IPA.

**Table 3:** Multidimensional logistic regression model evaluating BDG test quality and other possible influencing parameters in prediction of proven and probable IFD—galactomannan test excluded.

	Estimate *βi*	Std. error	*p*	e^*βi*^
Antifungals administration	− 2.300	1.845	0.2125	0.10026
BALF BDG	0.0179	0.005820	**0.0428**	1.0181
Serum BDG	0.002836	0.001454	0.0511	1.0028
Bacteria in BALF	− 0.4843	0.7061	0.4928	0.61613
Bacteremia	− 15.67	1125	0.9889	1.57 × 10^–7^
Absolute neutrophil count	− 0.2840	0.1721	0.0990	0.75277
Aspirated BALF volume	− 0.01266	0.01222	0.3002	0.98742
Interaction BALF BDG and BALF volume	− 0.0001286	0.00007475	0.0854	0.99987

BDG, 1,3-β-d-glucan; BALF, bronchoalveolar lavage fluid; IFD, invasive fungal disease; *βi*, regression coefficient; std. error, standard error; eβ, the chance that the episode will be closed as proven or probable IFD.

AIC = 150.66; χ^2^ = 31.38; R^2^ ≈ 0.19.

Bold value indicates statistical significance (*p* < 0.05).

Finally, our multivariate analysis, most consistent with real clinical practice, evaluated the quality of both BDG and GM diagnostic tests together during IPA diagnosis. Despite both tests being statistically significant, the GM test confirmed a 203-fold higher predictive value for serum and a 19-fold higher value for BALF compared to the BDG test (Table 4). Furthermore, there was correlation between higher BALF volume and reduced IPA predictive probability using the BDG test (*p* = 0.069) (Table 4).

**Table 4:** Multidimensional logistic regression model evaluating BDG test quality and other possible influencing parameters in prediction of proven and probable IFD—galactomannan test included.

	Estimate *βi*	Std. error	*p*	e^*βi*^
BALF BDG	0.02207	0.01210	0.068	1.02232
Serum BDG	0.003608	0.001535	**0.019**	1.00361
BALF GM	2.965	1.056	**0.005**	19.39470
Serum GM	5.315	2.464	**0.031**	203.36451
Absolute neutrophil count	− 0.2269	0.1725	0.188	0.797
Aspirated BALF volume	− 0.009625	0.01526	0.528	0.99042
Interaction BALF BDG and BALF volume	− 0.0003074	0.0001689	0.069	0.99969

BDG, 1,3-β-d-glucan; BALF, bronchoalveolar lavage fluid; GM, galactomannan; IFD, invasive fungal disease; *βi*, regression coefficient; std. error, standard error; e*βi*, the chance that the episode will be closed as proven or probable IFD.

AIC = 113.84; χ^2^ = 63.533; R^2^ ≈ 0.40.

Bold values indicate statistical significance (*p* < 0.05).

## Discussion

Our study substantiates efficacy of concomitantly obtained BALF and serum BDG samples from a large set of unselected consecutive hematological patients with pulmonary infiltrates for IFD diagnosis. BDG and GM values levels significantly correlated with each other in both serum and BALF (Fig. 1). We documented a substantially higher median of both BALF and serum BDG levels in episodes of proven and probable IFDs compared to no IFDs (*p* < 0.001). Moreover, the correlation between BALF or serum BDG levels and IFD probability was evidenced with statistical significance (*p* < 0.001; and *p* < 0.05; respectively) (Fig. 1). BDG displayed similar DOR in BALF compared to serum (6.5 vs. 6.2) at the same levels of SEN and SPE (57% and 83%). The BDG-max assay affirmed higher SEN (65%) but lower SPE (73%) and DOR (5.1).

BDG test quality is affected by a number of different factors. First, ***IFD definition*** may vary among studies. A vast majority of studies (including our investigation) did not include possible IFD in their analysis^23–25^. The quality of the BALF and serum BDG test did not significantly differ in the cohort with versus without 83 possible IFD in our analysis (DOR—6.9 vs. 6.5; and 4.2 vs. 6.2).

Additionally, ***degree and type of immunosuppression*** may facilitate BDG test accuracy discrepancies among studies. Our results are consistent with published data of Rose et al. and Theel et al., who reported BALF and serum BDG sensitivity in the range of 50–53%, and 40–55%, with a similar spectrum of hematological patients, respectively^23,25^. Most published studies used the same ***Fungitell cut-off level*** (≥ 80 pg/ml) for both serum and BALF as our study. He et al. in a large meta-analysis, reported serum BDG diagnostic accuracy and set the optimal cut-off level of 60 pg/ml as optimum for distinguishing patients with and without IFD^16^. Similar to He's study, we confirmed a lower optimal cut-off compared to the standard cut-off in both serum and BALF with better SEN and DOR, yet slightly lower SPE (Table 2).

### *Candida *spp. colonization or infection

Concomitant *Candida* spp. colonization or non-invasive respiratory infections may influence BALF BDG specimen false positivity and assay accuracy. Therefore, BALF BDG test SPE differences between published studies (39–68%) and our data (83%) could be caused by the variable frequency of *Candida* spp. positive cultures from BALF in episodes with no IFD compared to our study (32–43% vs. 6%)^23–25^. Furthermore, our study recognized a lower proportion of *Candida* spp. positive cultures from BALF in Fungitell positive episodes with no IFD compared to Theel's study (16–38%)^25^. Nonetheless, based on a multivariate analysis and in accordance with Rose's study^23^, we did not substantiate concomitant positive *Candida* spp. culture from BALF or oral cavity as a factor significantly affecting BALF BDG assay performance (Table 3).

### Bacterial infection

In concordance with published data, we confirmed BDG test lower predictive value in episodes with positive bacterial culture from BALF (*Enterococcus* sp., *Pseudomonas aeruginosa*, *Klebsiella* sp., *Streptococcus* sp.) or concomitant bacteremia (*Enterococcus* sp., *Klebsiella* sp.), although without statistical significance using the multivariate analysis (Table 3)^28–30^. As with our data, Rose's BALF BDG assay performance was not significantly affected by concurrent pulmonary bacterial infection^23^.

### Neutropenia

Published studies analyzing a BALF or serum BDG test in non-neutropenic patients or in cohorts with a lower proportion of neutropenic patients determined a lower SPE (26–65%) compared to our study^17–19,23–26^. Our analysis affirmed a higher neutrophil count during sampling as a factor adversely affecting BDG test performance within an IFD prediction with a statistical trend in significance (Table 3). To our knowledge, such a comprehensive analysis evaluating BDG test accuracy within an IFD diagnosis according to degree of neutropenia has not yet been conducted.

### Antifungal therapy

Antifungal therapy was significantly associated with false-negative BDG results in both BALF and serum in Rose's analysis and with serum in Ostrosky-Zeichner's study^20,23^. In contrast to published studies, we did not determine any significant relationship between antifungal administration or antifungal treatment duration and BALF or serum BDG assay performance in the multivariate analysis (Table 3). However, we are aware of our high proportion of episodes treated with antifungals at the time of cohort sampling reflecting clinical practice. For this reason, stricter criteria are set for BDG diagnostic test performance with IFD diagnosis.

### Sampling method and BAL standardization

Sampling method and timing could be another source of heterogeneity and affect fungal antigens detection accuracy in both BALF and serum. Theel et al. analyzed serum samples collected within 72 h of the BAL, which was at variance with our study sampling BALF and serum at precisely the same time^25^. Other investigations failed to note sampling time^23,24^. As predicted, specificity increased up to 100% by using two serum BDG test sequential positivities^31,32^.

BAL procedure standardization is a significant factor. Instilled solution during bronchoscopy ranging from 100 to 200 ml represents one of the crucial factors contributing to the final amount of aspirated BALF volume and consequently to BDG concentration and assay reactivity^21,25^. Notably, specific published data concerning BDG assay is lacking. Comparable to Racil's study evaluating the BALF GM test, our analysis affirmed better BALF BDG performance in patients with lower aspirated BALF volume (Tables 3, 4)^33^.

In our cohort, both BDG and GM antigens were simultaneously investigated from serum and BALF. In concordance with our results, a galactomannan test substantiated superiority to the BDG tested in both BALF and serum when diagnosing invasive pulmonary aspergillosis in literature^26,34^.

Our study highlights the advantage of BALF and serum BDG concomitant obtained samples including one of the largest published sets of non-selected consecutive hematological patients with pulmonary infiltrates relating to IFD diagnosis. Furthermore, our analysis precisely assesses predictable factors affecting BDG test accuracy.

Nonetheless, potential study limitations reflecting real clinical practice depict a low number of proven/probable IFDs, a high proportion of antifungal therapy at the time of sampling, and BALF specimen serum cut-off implementation without updated established criteria. Moreover, patients were only tested once with serum Fungitell assay. Although our study is a single-center analysis, it is a homogeneous, reproducible, and verifiable cohort.

## Conclusions

In conclusion, we confirmed acceptable SEN, SPE, DOR, and a low PPV of both BALF and serum BDG assay for pulmonary IPA diagnosis. Furthermore, we recorded high NPV for both BALFs and sera, predisposing a basic BDG assay utility to exclude IFDs indicating serum value with patient IFD screenings. BDG test sensitivity and DOR were not substantially increased by BALF compared to sole serum testing, and their combination did not improve test quality. Dedicated efforts to determine optimal BDG limit value did not facilitate significant test quality improvement.

We confirmed that: (1) absolute neutrophil count at the time of sampling and (2) aspirated BALF volume both considerably affect assay performance.

Consequently, although BALF and serum GM and BDG values correlated with each other, BDG continued to reveal reduced test quality compared to GM in IPA diagnosis. Our study fully supports the recent Infectious Diseases Society of America (IDSA) and EORTC/MSG recommendations to avoid using BDG for defining IFD and to restrict use to specific clinical settings in conjunction with other clinical findings.
